# Supplementary material for: Correlates of tuberculosis in children under the age of 15 years
Source: PLoS One. 2025 Feb 12;20(2):e0312570. doi: 10.1371/journal.pone.0312570 (PMC11819535; doi:10.1371/journal.pone.0312570)
Supplement: Attachment — Submitted filename: RESPONSE TO REVIEWERS.pdf [file pone.0312570.s003.pdf]

Mulungushi University School of Medicine and Health Sciences
HAND Research Group
P.O Box 60009, Livingstone Campus.

Email:smasenga@mu.ac.zm website:www.mu.ac.zm Contacts: +260 966674774

20th June, 2024

To the Editor and Reviewers,

RESPONSES TO EDITORIAL AND REVIEWER COMMENTS

When submitting your revision, we need you to address these additional requirements.

1. Please ensure that your manuscript meets PLOS ONE's style requirements, including those for file naming. The PLOS ONE style templates can be found at https://journals.plos.org/plosone/s/file?id=wjVg/PLOSOne_formatting_sample_main_body.pdf and https://journals.plos.org/plosone/s/file?id=ba62/PLOSOne_formatting_sample_title_authors_affiliations.pdf

Response: done

2. Please provide additional details regarding participant consent. In the ethics statement in the Methods and online submission information, please ensure that you have specified (1) whether consent was informed and (2) what type you obtained (for instance, written or verbal, and if verbal, how it was documented and witnessed). If your study included minors, state whether you obtained consent from parents or guardians. If the need for consent was waived by the ethics committee, please include this information.

If you are reporting a retrospective study of medical records or archived samples, please ensure that you have discussed whether all data were fully anonymized before you accessed them and/or whether the IRB or ethics committee waived the requirement for informed consent. If patients provided informed written consent to have data from their medical records used in research, please include this information.

Response: done

3. We note you have included a table to which you do not refer in the text of your manuscript. Please ensure that you refer to Table 2 in your text; if accepted, production will need this reference to link the reader to the Table.

Response: done

4. Please include captions for your Supporting Information files at the end of your manuscript, and update any in-text citations to match accordingly. Please see our Supporting Information guidelines for more information: http://journals.plos.org/plosone/s/supporting-information.

Mulungushi University School of Medicine and Health Sciences
HAND Research Group
P.O Box 60009, Livingstone Campus.
Email:smasenga@mu.ac.zm website:www.mu.ac.zm Contacts: +260 966674774

Response: done

REVIEWER #1

It would be helpful to have line numbers for ease of reference when reviewing the manuscript.

Response: thank very for the suggestion. We have added line numbers to the manuscript.

1. This sentence in the introduction is not accurate and must be revised "According to the World Health Organization (WHO), an estimated 10 million people worldwide were infected with tuberculosis in 2020", It developed and not infected. Almost 1/3 of the global population are infected with TB.

Response: thank you very for the correction. The sentence has been revised to imply development of cases instead of infections.

2. Please check reference 5, I don't think it is the correct reference. Going through the article, there is no indication or discussion about prevalence of TB. Also, prevalence of TB of 12% and 15% in Malawi and South Africa seems very high. Check that you have the correct interpretation of this. Many high burden countries have a prevalence of around 1% of TB, it is lower in children. Perhaps the authors wanted to say that "out of all TB notification, children accounted for 12 and 15% etc"

Response: thank very for the correction. The sentence has been revised with new accurate information.

3. The reference for the first sentence in paragraph 3 of the introduction session is not the best reference for this and it is old (2016). There are newer references for this. For example WHO list of high burden countries?

Response: thank very for the correction. The sentence has been revised with latest data from WHO report of 2023 Global tuberculosis report 2023.

4. The first sentence of paragraph 3 needs to be revised for clarity based on the list of high burden countries

Response: thank very, the sentence has been revised and clarified

5. Consider revising this sentence "A recent national survey conducted by kapata et al (2016) estimated the prevalence of 455/ 100000 persons for a population aged 15 years and older and the burden was high among people living with HIV (PLWH) (10)"

Mulungushi University School of Medicine and Health Sciences
HAND Research Group
P.O Box 60009, Livingstone Campus.
Email:smasenga@mu.ac.zm website:www.mu.ac.zm Contacts: +260 966674774

This survey was conducted in 2013/ 2014, publication was 2016, therefore it is not recent, perhaps, the last survey conducted in xx years found.

Response: thank very for the suggestion. The sentence has been updated with current incidence as of 2022 published by Health press bullet in 2022 and WHO global tuberculosis report 2023.

6. What guided or what assumptions were used to set 50% prevalence of TB? That is half the population with TB. Did the authors get help from an epidemiologist or a biostastician to do the sample size calculations

Response: thank you very much. We originally used 50% in order to generate the highest maximal sample size so that our study is highly powered. However, we have now recast and recalculated the sample size with help from a biostatistician. We have used a prevalence of 9 % of active TB case recorded in Zambia. Our study is still highly powered with a current sample size above the minimal required. We thank the reviewer for highlighting this.

7. The authors tend to mix tuberculosis infection with active TB disease, this needs to be corrected through the manuscript. Treatment for tuberculosis infection is not the same as treatment for active TB disease.

Response: thank very. We have modified this throughput the manuscript.

8. Clarify if "Data were collected by three (3) trained research assistants who abstracted data from patient files between August 1, 2023 and January 8th, 2024." this means only for patients treated during this time period were sampled. And what guided the selection of these dates.

Response: thank very. "Data were collected by three (3) trained research assistants who abstracted data from patient files between August 1, 2023 and January 8th, 2024." Was meant to show the period when data abstraction was done. We abstracted children's files who were treated between 1st January 2020 to 31 December 2022. We have now clarified this in the manuscript' methods section. Thank you for bringing up this.

9. Data analysis- more detail is needed on how regression analysis was done, especially how the model was built to decide on the final variables for inclusion in the model.

Response: thank very. We have clarified how the model was built. The regression was built based on the variables which had a p value less than 0.05.

Results section

10. Why use the word about, when you have an actual measure "about 9.2 people were living with HIV....."

Response: thank very. The sentence has been corrected.

11. Table 1 is a bit confusing. Suggest to present column % in columns 3 and 4 and not row %s

Response: thank very for the suggestion. Column % have now been used in table one.

Discussion

12. First line in the discussion is again mixing up TB infection and TB disease

Response: thank very much for the correction. We have now corrected this and all other instances where we mixed up TB infection and TB disease

13. Second paragraph 1st sentence needs to be specific to indicate that the finding is among hospitalised children. As it is written, it gives the impression that this finding is in the general population.

Response: thank very for the suggestion. We have corrected

14. For this sentence "This prevalence rate is slightly different with previous studies conducted in Sudan which was 15.3% and other studies have estimated it to be approximately 11% (12,13)." Check and be sure you are comparing similar populations. i.e hospitalized children

Response: thank you very much for the correction. The studies have been changed to reflect the populations being compared

15. In the discussion, you might want to also reference the Zambian postmortem studies by Chintu et al, Mudenda et al

Response: thank very for the suggestion. The study of mudenda et al has been cited as it was conducted in-patients

REVIEWER #2:

please state How many years record was reviewed

Response: thank very. The data reviewed was a period of 3 years (between 1st January 2020 to 31 December 2022) we have now included this in the design and setting of the methods section.

Was the data a clinic data, admissions or total number of children seen in the hospital and over what specified period?

Mulungushi University School of Medicine and Health Sciences

HAND Research Group

P.O Box 60009, Livingstone Campus.

Email:smasenga@mu.ac.zm website:www.mu.ac.zm Contacts: +260 966674774

Response: thank very. The data was admission of children over a period of 3 years (between 1st January 2020 to 31 December 2022) we have included in the design and setting in the methods section.

The manuscript is relevant, but they need to state how many years data was the review ie period

Percentage of HIV 9.1 % or 9 %

Response: thank very. The number of years data was reviewed has been stated now in the methods. The prevalence of HIV among the children was 9.2% (62/675).

LIMITATION. One of the limitation the number of children of 66 out of 700 with tuberculosis was small and diificult to interprete , however this is possibly how many were available to them , they need to state where the patient were from ,refered or patient within the community ie scope of the center where the study was carried out

Response: thank very. We have included your suggestion as one of the limitations and also included details of the study population

We thank you all for all your valuable suggestions that has improved our manuscript. We now hope it is suitable for publication. We look forward to hearing from you at your earliest convenience. All authors have approved the final version.

Yours sincerely,

Prof. Sepiso K. Masenga
