# Supplementary material for: Correlates of tuberculosis in children under the age of 15 years
Source: PLoS One. 2025 Feb 12;20(2):e0312570. doi: 10.1371/journal.pone.0312570 (PMC11819535; doi:10.1371/journal.pone.0312570)
Supplement: Attachment — Submitted filename: Reviewer response letter.pdf [file pone.0312570.s004.pdf]

Mulungushi University School of Medicine and Health Sciences
HAND Research Group
P.O Box 60009, Livingstone Campus.
Email:smasenga@mu.ac.zm website:www.mu.ac.zm Contacts: +260 966674774

15th September 2024
To the Editor,

Dear Editor,

Ref: Reviewer responses

The above matter refers.

We would like to thank the reviewer for their invaluable suggestions to further improve our manuscript. We have revised the manuscript accordingly. We hope it is now satisfactory. Below are the specific changes and responses.

Reviewer #1: Line 1:
Use updated TB report. The latest is 2022

Response: thank you for the correction, the statistics have been updated as suggested

Line 94:
Sample size calculation is still an issue. I think the authors are using the wrong reference for this. In what population is this estimate from? It is incorrect to use the proportion of children notified as the prevalence. I think this is what the authors did but I may be wrong. The authors needed to have used any data available on prevalence of TB among hospitalised children. What about postmortem studies from Zambia, what did that show.

Response: thank you very much the suggestion. We have recalculated the sample size using the 2016 bates et al postmortem studies among hospitalized pediatric patients who died. Which revised the prevalence to 8 % active TB.

Line 142 Regression analysis, further description is needed here. How was the final model arrived at? Was it backward or forward addition? Why are the authors reporting unadjusted ORs. Unj OR can be due to chance occurrence? Adjusted ORs is what is reported. So only HIV and Malnutrition were associated with TB. But the authors need to advise how the regression analysis was done.

Response: Thank you very much. We have reanalyzed the data using the forward stepwise Generalized Linear Models equations (GLM) via the logit link function to account for correlation structure within observations and to select the most significant predictors that were included in the final model. We first reported the unadjusted ORs just to show that it was the first step we did to build the model; however, the unadjusted ORs were not discussed in the discussion due to potential confounders.

Mulungushi University School of Medicine and Health Sciences
HAND Research Group
P.O Box 60009, Livingstone Campus.
Email:smasenga@mu.ac.zm website:www.mu.ac.zm Contacts: +260 966674774

Line 232: The authors need to be clear that they are referring to hospitalised children and should not generalised this finding. This is a highly selected group so this sentence needs to mention this.

Response: we have corrected as suggested. Thank you

Conclusion: Is generalized. Needs to be specific that this is in hospitalized children.

Response: thank you for the correction. The sentence has been corrected

Line 238 perhaps overestimate?
Response: thank you. We have substituted underestimate with overestimate as suggested.

Conclusion: Is generalized. Needs to be specific that this is in hospitalized children.
Response: thank you for the concern. We have now specified the population is among hospitalized children at our facility

Once again, we thank you for your support in helping to improve our manuscript

We look forward to hearing from you at your earliest convenience.

Yours sincerely,

Prof. Sepiso K. Masenga
